# Temporal and Spatial Heterogeneity of PM_2.5_ Related to Meteorological and Socioeconomic Factors across China during 2000–2018

**DOI:** 10.3390/ijerph19020707

**Published:** 2022-01-09

**Authors:** Xiangxue Zhang, Changxiu Cheng

**Affiliations:** 1 Key Laboratory of Environmental Change and Natural Disaster, Beijing Normal University, Beijing 100875, China; zxx@lreis.ac.cn; 2 State Key Laboratory of Earth Surface Processes and Resource Ecology, Beijing Normal University, Beijing 100875, China; 3 National Tibetan Plateau Data Center, Beijing 100101, China

**Keywords:** PM_2.5_ pollution, spatiotemporal heterogeneity, Northern and Southern China, GeoDetector, impact factors

## Abstract

In recent years, air pollution caused by PM_2.5_ in China has become increasingly severe. This study applied a Bayesian space–time hierarchy model to reveal the spatiotemporal heterogeneity of the PM_2.5_ concentrations in China. In addition, the relationship between meteorological and socioeconomic factors and their interaction with PM_2.5_ during 2000–2018 was investigated based on the GeoDetector model. Results suggested that the concentration of PM_2.5_ across China first increased and then decreased between 2000 and 2018. Geographically, the North China Plain and the Yangtze River Delta were high PM_2.5_ pollution areas, while Northeast and Southwest China are regarded as low-risk areas for PM_2.5_ pollution. Meanwhile, in Northern and Southern China, the population density was the most important socioeconomic factor affecting PM_2.5_ with *q* values of 0.62 and 0.66, respectively; the main meteorological factors affecting PM_2.5_ were air temperature and vapor pressure, with *q* values of 0.64 and 0.68, respectively. These results are conducive to our in-depth understanding of the status of PM_2.5_ pollution in China and provide an important reference for the future direction of PM_2.5_ pollution control.

## 1. Introduction

In the past few decades, the acceleration of urbanization has affected air quality, human and ecological health worldwide [1,2,3]. In China, rapid economic development and the increase in energy-intensive industries have caused serious environmental pollution problems [1,4]. In particular, of the various atmospheric pollutants, particulate matter with an aerodynamic diameter of less than 2.5 μm (PM_2.5_) is the most harmful [5,6], which can severely impact environmental conditions, visibility and driving conditions, and climate change [3,7]. In Beijing, annual PM_2.5_ concentrations exceeded the Chinese National Ambient Air Quality Standard Level 2 (35 μg/m^3^) at all sites in 2014, especially at traffic sites, where annual average values were as high as 98.6 ± 89.0 μg/m^3^ [8]. Annual PM_2.5_ concentrations in China ranged from 10 to 157 μg/m^3^ in 2016, with 78.79% of the total population exposed to PM_2.5_ concentrations > 35 μg/m^3^. Subsequently, the national PM_2.5_ mortality rate was 0.96 (95% confidence interval (CI): 0.45, 13.55) million, accounting for approximately 9.98% of the total reported deaths in China [9]. In China, we estimate that 12,100 Chinese people (95% CI: 10,300–13,800) would die from PM_2.5_ exposure each year [10]. More worryingly, some previous studies reported that with the rise in PM_2.5_ concentration, the mortality rate of cardiovascular and respiratory diseases is also increasing [11,12,13].

The severity of PM_2.5_ pollution is well accepted to be strongly related to anthropogenic emissions [14], topography [15], meteorology [16,17], and physical and chemical reactions [18,19]. Due to its rapid urbanization and urban agglomerations, air pollution episodes have been frequented in China. However, the speed and stages of urbanization significantly differ among cities, which experience varying degrees of environmental problems and lead to spatial differences in PM_2.5_ concentrations [20]. More seriously, China faces the highest mortality rate associated with PM_2.5_ exposure worldwide [21], meaning that severe PM_2.5_ pollution has had an impact on human health; therefore, it has caused concern in many fields. Notably, meteorological and socioeconomic variables are important external factors that can impact a city’s accumulation and diffusion of pollutants. Therefore, the association between meteorological and socioeconomic factors and PM_2.5_ across China was investigated in order to further realize the sustainable development of cities.

For example, in 68 major cities in China, Yang et al. [22] reported the linear relationship between PM_2.5_ and meteorological factors, and found that this connection has temporal and spatial changes. Chen et al. [23] analyzed the relationship between meteorological factors and air pollution in Nanjing, finding a negative association between PM_2.5_ and temperature, wind speed, precipitation, and relative humidity. Similarly, in the Sichuan Basin, Li et al. [24] found a negative relation between PM_2.5_ concentrations and wind speed, temperature, and precipitation, while the PM_2.5_ concentration had a positive correlation with air pressure. Li et al. [25] showed that an inverted V-shaped characteristics were found between PM_2.5_ concentration and temperature, and PM_2.5_ was negatively correlated with precipitation, wind speed, and relative humidity.

Furthermore, PM_2.5_ concentrations in China show clear spatial heterogeneities. Some studies have shown that PM_2.5_ pollution is closely correlated to socioeconomic factors because rapid urbanization and economic development are associated with those activities linked to PM_2.5_ emission, because rapid urban development would lead to increased industrial activities, attract large population concentrations, increase road density and biomass burning, and, therefore, contributions to the spatial distribution in PM_2.5_ concentration. For example, Wang et al. [26] showed that population density significantly impacts PM_2.5_ pollution, and Yang et al. [27] found that road density had a high impact on PM_2.5_ concentrations. In addition, Zhu et al. [28] presented that the PM_2.5_ pollution in urban areas was more severe than in the surrounding regions, demonstrating that urbanization has a non-ignorable impact on PM_2.5_ pollution. Li et al. [29] demonstrated that, especially in metropolitan areas, the impact of PM_2.5_ exposure levels on dynamically changing population movements varies from time to time.

Previous studies have rarely calculated the effects of the interactions of influencing factors on PM_2.5_ based on spatially stratified heterogeneity. Moreover, traditional statistical methods to calculate the interactions of factors can only calculate the product of two factors, and they have poor ability to explain spatially stratified heterogeneity. However, the interaction of two factors can be coupled in many forms in reality; therefore, through this study, our goal is to: (1) understand the long-term changes of PM_2.5_ pollution in China and its temporal trends and spatial heterogeneity; (2) analyze the high and low-risk areas (indicating that probability of a region becoming more/less polluted relative to the average PM_2.5_ concentrations of the study area) of PM_2.5_ pollution-related mechanisms; and (3) expose the determinant meteorological and socioeconomic factors and their interactive impacts based on GeoDetector for identifying regional patterns of PM_2.5_ in Northern and Southern China.

The study of PM_2.5_ pollution and its influencing factors on a long historical time series and a large spatial scale can comprehensively provide an important direction for future PM_2.5_ management and can provide a reference for the formulation of policies.

## 2. Methods

### 2.1. Region and Data

**Figure 1:**
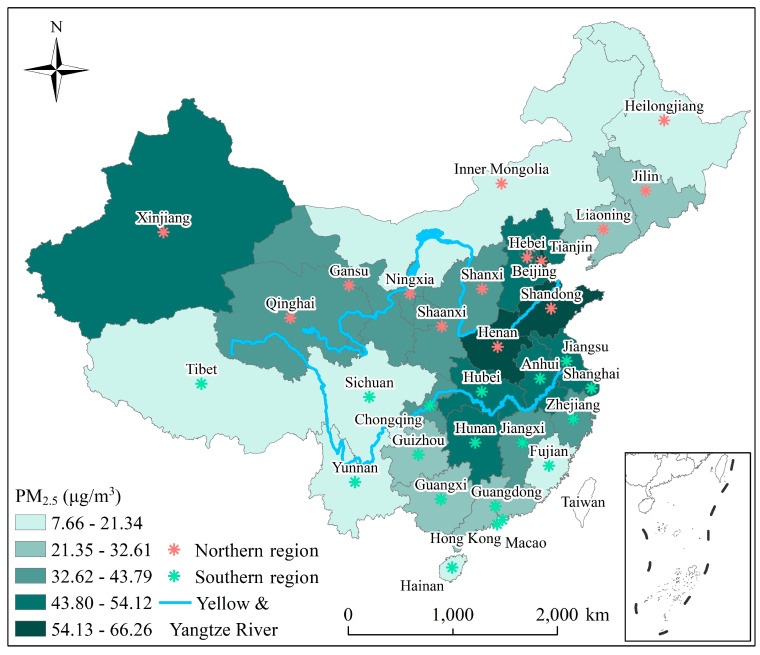
Spatial distribution of annual mean PM_2.5_ concentrations (2000–2018) across China.

There is a significant climatic variation from the coast to inland areas in China due to geographical effects. According to the Ministry of Environmental Protection of China, air quality in Baoding, Handan, Jinan, Langfang, Hengshui, Shenyang, Shijiazhuang, Tangshan, Xingtai, and Zhengzhou, in 2015, was poorest [30], all of which are located in Northern China. Given the reference from Zhang et al. [31], China has been divided to two regions to further analysis the studied question; therefore, in our analyses, we also broadly divided China into the following two regions: (1) Northern (Heilongjiang, Liaoning, Jilin, Gansu, Ningxia, Inner Mongolia, Xinjiang, Qinghai, Beijing, Shanxi, Hebei, Tianjin, Henan, Shaanxi, and Shandong) and (2) Southern (Jiangsu, Anhui, Shanghai, Hubei, Guangxi, Chongqing, Sichuan, Tibet, Yunnan, Guangdong, Hainan, Zhejiang, Fujian, Jiangxi, Hunan, and Guizhou) (Figure 1).

**Figure 2:**
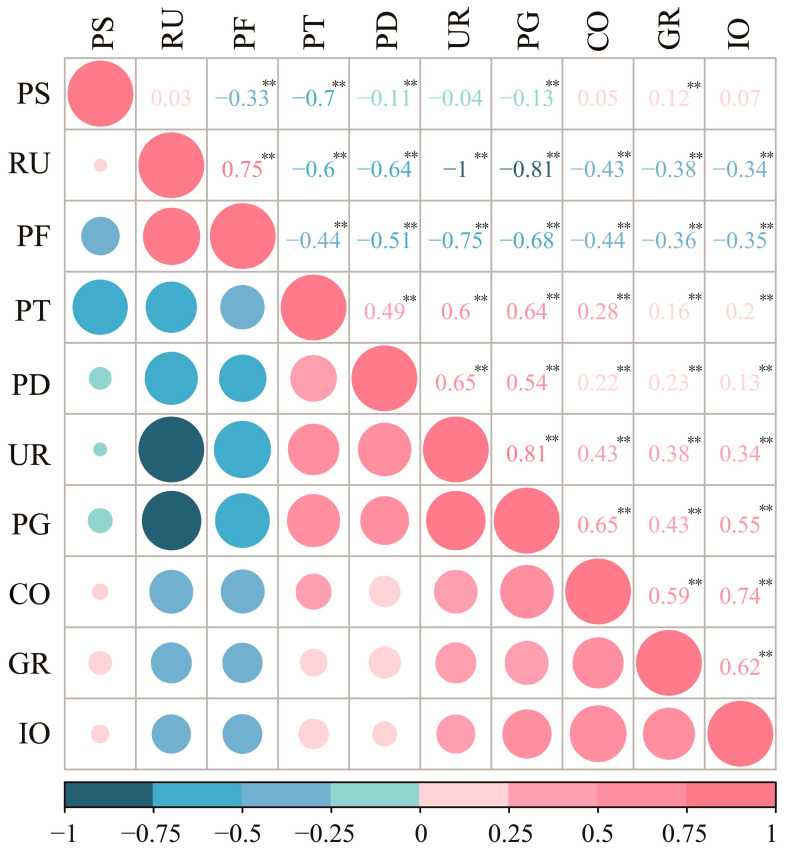
Correlation matrix for socioeconomic factors. Note: PD: population density; UR/RU: the non-agricultural/agricultural proportion of the population; PG: per capita gross domestic product; IO: industrial output; PF/PS/PT: proportion of primary/secondary/tertiary industry; CO: construction output; GR: area of green land. ** statistical significance level: 0.01.

Data on PM_2.5_ from 2000 to 2018 were obtained from a group of Dalhousie University, that is, https://sites.wustl.edu/acag/, which was accessed March 2021. It (0.01° × 0.01°) offers high accuracy, having been verified, and the R^2^ value was 0.81 [32]. Then, we have extracted the annual mean values of PM_2.5_ for each province to further analysis. Meteorological explanatory variable data downloaded from the China Meteorological Data Sharing Service System refers to average temperature (AT), vapor pressure (VP), wind speed (WS), relative humidity (RH), and precipitation (PC). Annual socioeconomic data for 31 provinces refers to population density (PD); the non-agricultural proportion of the population (UR); the agricultural proportion of the population (RU); industrial output (IO); per capita GDP (hereafter PG); construction output (CO); proportion of the primary industry (PF); proportion of the secondary industry (PS); proportion of the tertiary industry (PT); and the area of green land (GR) were obtained from the Chinese governmental statistical yearbooks. Given the strong correlations between socioeconomic factors (Figure 2) and the results from other studies, PD, UR, PG, IO, PS were ultimately selected for inclusion in the study. Because PM_2.5_ historical dataset used in the study is annual data, i.e., there is one PM_2.5_ value for each region each year, the annual average values of impact factors are used in the calculation to ensure that the dependent and independent variables are consistent on the time scale.

### 2.2. Statistical Methods

#### 2.2.1. Bayesian Space–Time Hierarchy Model (BSTHM)

BSTHM is a statistical method applied to investigate the representative spatiotemporal patterns of the spatiotemporal data. Second, BSTHM can also effectively solve the problem of small sample size in spatiotemporal data and help to show the interpretation of spatiotemporal correlations [33]. Third, it can solve the complex spatiotemporal coupled stochastic processes. Moreover, BSTHM provides more information than traditional techniques in spatiotemporal analysis, which has been applied in many subjects [34,35,36]. Thus, this method in the study was used to detect the spatial–temporal heterogeneity of PM_2.5_ pollution.

Here, the PM_2.5_ data (2000–2018) for the 31 study provinces were used as the spatiotemporal data, as follows:

(1) $$y_{it}∼N\;\left(u_{it},\;\sigma_{y}^{2}\right)$$

(2) $$log\left(u_{it}\right)=\alpha +s_{i}+\left(b_{0}t^{*}+v_{t}\right)+b_{1i}t^{*}+\varepsilon_{it}$$

where *y_it_* is the average PM_2.5_ of province *i* (*i* = 1, 2, ..., 31) in year *t* (*t* = 1, 2, ..., 19), *u_it_* stands for the overall potential pollution risk during the study period, *σ_y_^2^* is the variance, assuming that these items conform to normal distribution, *α* represents the overall log pollution risk over the 19 years in China [33], *s_i_* represents the spatial characteristics of the PM_2.5_ risk during 2000–2018, (b_0_t^*^ + *v_t_*) indicates pollution risk’s temporal variation, t^*^ is observation period calculating from the mid-study period, *b_1i_* is the local temporal trend relative to the overall trend *b_0_*, and *ε_it_* ~ *N* (0, *σ_ε_^2^*) is the Gaussian noise [37].

According to the following two-step process, proposed by Richardson et al. [38] pollution high/low-risk regions (and neither) were determined. First, when the posterior probability *p* (*exp* (*s_i_*) >1|data) exceeds 0.80 or is lower than 0.20, respectively, it is defined as a high-risk or low-risk region. Those provinces falling between these values were neither defined as high-risk region nor low-risk region. Here, exp (*s_i_*) represents the average pollution risk (overtime) in province i relative to α, the China average [33]. Second, referring to the posterior probability of local slope *exp* (*b_1i_*), each of these categories was further classified; that is, a faster or slower increasing trend corresponding to the overall trend was assumed when *p* (*b_1i_* > 0|*h_i_*, data) ≥ 0.80 or ≤ 0.20, respectively; if local trend approximated the overall that where 0.20 < *p* (*b_1i_* > 0|*h_i_*, data) < 0.80. Notably, increasing and decreasing trends are relative to a common trend and, therefore, do not necessarily correspond to an absolute increase/decrease in pollution risk [33]. Here, we can obtain nine categories (i.e., risk categories (three) × trend categories (three)), which are calculated in WinBUGS 14 [39], a software designed for Bayesian calculation.

#### 2.2.2. GeoDetector

The GeoDetector, a non-linear method, (www.geodetector.cn (accessed on 16 August 2021)) can effectively quantify phenomena with spatially stratified heterogeneity whereby, assuming that impact factor X has some effect on the dependent variable Y and is statistically significant, then Y and X will have a similar distribution in space [40,41]. Moreover, GeoDetector has no linearity assumptions on the variables, which is one of its advantages. Therefore, it is possible to disregard the multicollinearity of the influencing factors, i.e., adding new factors or removing existing factors will not affect the results of other factors, as follows:

(3) $$q=1-\frac{1}{N\sigma^{2}}\sum_{h=1}^{L}N_{h}\sigma_{h}^{2}$$

where *q* (ranging [0, 1]) quantifies the effects of X or Xs on Y; *h* is the stratification of X or Xs according to its or their spatial characteristics and *h* = 1, 2, ..., *L*, and *L* is the number of strata [40,41]; *N* (is equal to 31) and *σ^2^*, in this study, denote the total number and variations of study units, respectively; corresponding, *N_h_* and *σ_h_^2^* are the numbers and variations of that in strata *h*, respectively.

Here, the GeoDetector, quantifying the effects of X and Xs (interaction between X1 and X2) on the Y (PM_2.5_ concentrations) and further calculating whether the effects of interactions (X1∩X2) between different factors on *Y* increases or decreases compared to the effects of X1 and X2 individually [40], is more comprehensive than traditional methods in calculating interactions, with richer results and better integration and consideration of real geographic features.

## 3. Results

### 3.1. Spatiotemporal Heterogeneity

**Figure 3:**
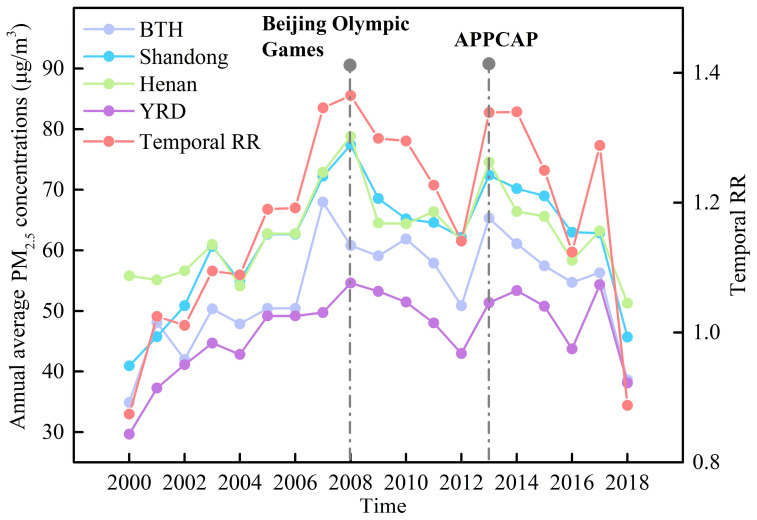
Temporal relative risks and variations in PM_2.5_ concentrations in different regions. Note: BTH is Beijing–Tianjin–Hebei region; YRD is Yangtze River Delta region; RR is relative risks; APPCAP is the Air Pollution Prevention and Control Action Plan.

Figure 3 depicts the overall temporal relative risks (RRs) in China during 2000–2018 and their temporal variations. Overall, a first increasing then decreasing trend was identified. It is worth noting that significant regional differences have also been identified in addition to temporal changes. Moreover, during two major events and policies (the Beijing Olympic Games (in 2008) and the Air Pollution Prevention and Control Action Plan (APPCAP) implemented by the Chinese government to improve the quality of the air environment from 2013 to 2017), the PM_2.5_ also presented temporal differences. In the Beijing–Tianjin–Hebei (BTH) region, the PM_2.5_ concentrations showed a first upward and then downward trend in the early and after 2018 Beijing Olympic Games, with a peak of 67.96 μg/m^3^, then showed a decreased trend after the APPCAP implemented in 2013. The PM_2.5_ concentrations in Henan and Shandong provinces continued to rise until 2008, reaching the highest values of 78.81 and 77.38 µg/m^3^, respectively. It showed a decreasing trend during the emission reduction phase of the 2008 Olympic Games, and a continuous decreasing trend after enacting the enactment of the APPCAP policy in 2013. In the Yangtze River Delta (YRD), PM_2.5_ concentrations continuously increased until 2008 with a peak value of 54.60 μg/m^3^, and then showed interannual fluctuations thereafter.

**Figure 4:**
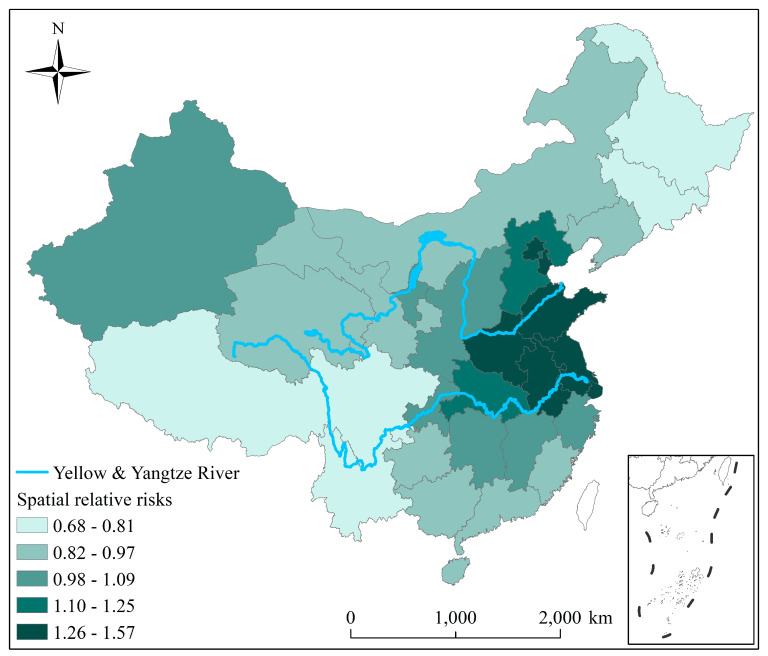
Spatial relative risks of PM_2.5_ concentrations in each province across China.

Spatially, the spatial RRs at the provincial-level from 2000 to 2018 exhibit notable spatial differences, demonstrated by the *q* value of 0.71 (Figure 4). The spatial RRs in major provinces of North China Plain (NCP) and YRD were higher, suggesting a severe PM_2.5_ risk in these areas, while pollution risks in Heilongjiang, Jilin, Tibet, and Yunnan are relatively low.

**Figure 5:**
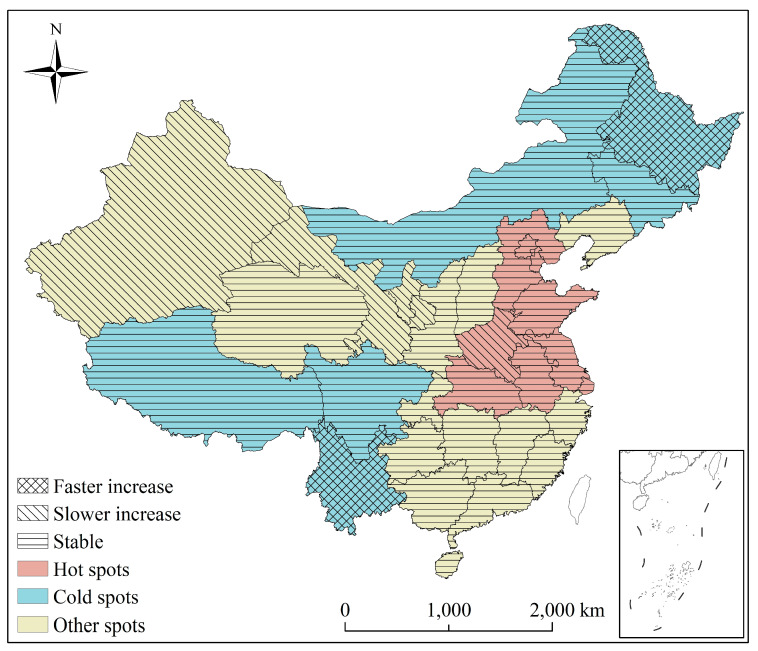
Spatial distribution of high/low-risk regions of PM_2.5_ concentrations in China.

Among the 31 provinces, nine (29.03%) and six (19.35%) were regarded as high and low pollution risk regions (high/low-risk region), respectively; the other 16 (51.61%) provinces did not belong to either classification (Figure 5).

Specifically, among the nine high risk regions, Henan showed a slower increasing trend than the overall. Thus, the risk in this region is likely higher, and the region will remain high risk in the future, deserving further attention (Figure 5).

Among the six low-risk regions, Heilongjiang and Yunnan showed a faster increasing trend compared to the overall increasing trend, indicating that the risks in the two provinces will be higher in the future. On the other hand, compared to the overall trend, Tibet, Sichuan, Inner Mongolia, and Jilin were consistent. Therefore, the risk in these spots is expected to remain relatively constant in the future (Figure 5).

Among those provinces that were not classified as high risk region or low-risk regions, Xinjiang, Gansu, and Ningxia showed a slow increasing trend; thus, these provinces will be high risk region in the future. Based on this, relevant departments in these provinces should be made aware of these potential risks. Moreover, the trends in Qinghai, Shanxi, Shaanxi, Liaoning, Chongqing, Guizhou, Hunan, Jiangxi, Zhejiang, Fujian, Guangdong, Guangxi, and Hainan, were similar to the overall trend (Figure 5), suggesting that PM_2.5_ pollution in these provinces will remain relatively stable in the future.

### 3.2. Impact Factors Analysis

Based on the GeoDetector, the highest factors affecting PM_2.5_ concentrations were average temperature and population density in Northern China and vapor pressure and population density in Southern China, as shown in Table 1 and Table 2.

Among the selected meteorological factors, AT and VP had the greatest impact on the temporal heterogeneity of PM_2.5_ concentrations (*q =* 0.64 and 0.68) in Northern and Southern China, respectively. Meanwhile, the *q* value of WS was not significant in Northern China (Table 1).

Socioeconomic factors were also found to play an important role. Shown in Table 2, whether in Northern or Southern China, the effect of PD on PM_2.5_ pollution was strongest (*q* = 0.62 and 0.66, respectively), meanwhile, the *q* values for PS and IO were not statistically significant (Table 2).

These results showed that both meteorological and socioeconomic factors significantly impacted the pattern of PM_2.5_ concentrations in China.

**Figure 6:**
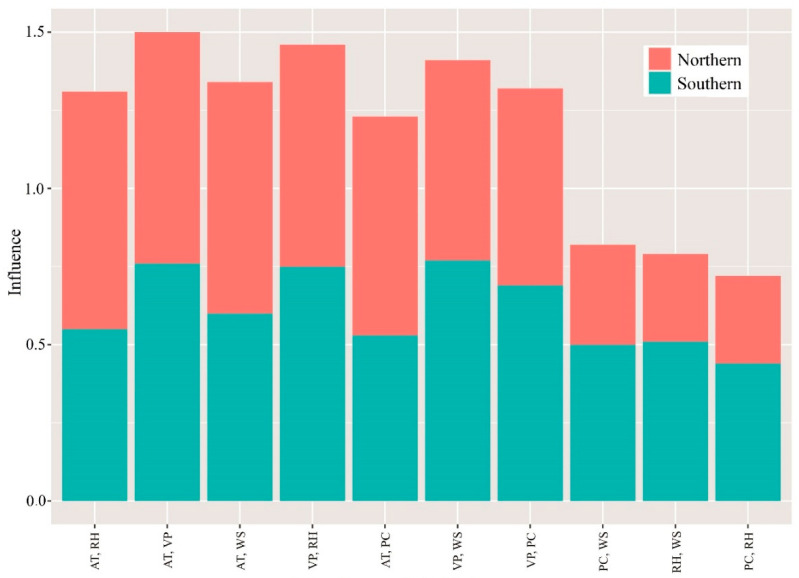
Interactions among meteorological factors in Northern and Southern China. Note: AT: average temperature; VP: vapor pressure; PC: precipitation; RH: relative humidity; WS: wind speed.

**Figure 7:**
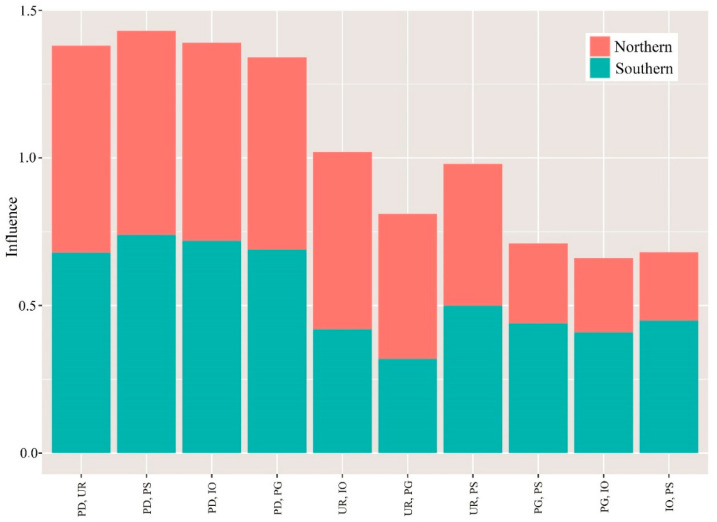
Interactions among socioeconomic factors in Northern and Southern China. Note: PD: population density, UR: the non-agricultural proportion of the population, PG: per capita gross domestic product, IO: industrial output, PS: proportion of secondary industry.

Most of the previous studies have rarely calculated the impacts of the interactions on PM2.5 considering spatially stratified heterogeneity. Moreover, traditional statistical methods have a poor ability to explain spatially stratified heterogeneity; therefore, the interactive influencing powers of combinations of the ten different meteorological or socioeconomic factors were quantified by the GeoDetector in this study. Importantly, the effects of these interactions for each pair of meteorological and socioeconomic factors were much higher than their individual effects, highlighting the importance of interactive effects on PM_2.5_ pollution (Figure 6 and Figure 7), and indicating that the interaction of factors enhances the effect of individual factor on PM_2.5_ pollution. Furthermore, in different regions, the dominant factors also vary.

Specifically, the strongest interactive effect of meteorological factors gave a *q* value of 0.76 for both AT and RH, and AT and VP, in Northern China; the strongest interactive effect with a *q* value of 0.77 for VP and WS in Southern China (Figure 6), indicating that one meteorological factor combined with other meteorological factors had a stronger influence on PM_2.5_ pollution.

As for socioeconomic factors, in Northern China, the strongest interactive effect with a *q* value of 0.70 for PD and UR; and PD and PS with a *q* value of 0.74 in Southern China (Figure 7), denoting that population activities interacted with other socioeconomic factors, such as urbanization and industrial activities, would enhance their effect on PM_2.5_ pollution.

## 4. Discussion

As shown in the above results, we observed significant spatial heterogeneity in the risk of PM_2.5_ pollution, the high-risk regions were mainly distributed in the North China and YRD regions. Socioeconomic conditions are crucially important here, and they are regarded as important drivers of PM_2.5_ pollution. For example, PD showed the highest association with PM_2.5_ risk in both Northern and Southern China, which can be demonstrated from previous research [27,35]. Ding et al. [42] also demonstrated that PD is the most strongly associated with PM_2.5_ concentrations in China, which validates our results. Similar results were found in the study of Lou et al. [43], who indicated that PD strongly affects PM_2.5_ concentrations in the Yangtze River Delta. However, previous studies have mainly focused on the effect of single factors; the interactive effect of impact factors are rare, as shown in this study, and the interaction of PD with other factors such as UR and PS has a more significant impact on PM_2.5_. This reflects that emissions from human activities largely affect PM_2.5_ concentrations and, when combined with other factors, greatly enhance the impact of individual factors on PM_2.5_ concentrations. The association is linked to anthropogenic emissions of secondary aerosols [44]. Furthermore, as in recent decades, China has experienced rapid urbanization, the continuous expansion of cities enhances the urban heat island effect, further promoting the generation and accumulation of pollutants and associated environmental hazards. Moreover, high-density populations, especially in the big cities, consume greater amounts of non-renewable energy, which is considered the main source of PM_2.5_ [45].

Urbanization was also spatially related to PM_2.5_ concentrations, especially in Northern China. Notably, China is undergoing rapid urbanization, and the resulting impact on PM_2.5_ pollution cannot be ignored. In North China, using remote sensing data, Zhang et al. [35] also found a positive correlation between urbanization and PM_2.5_ concentration, validating our results. Similar results were found in the study by Yang et al. [20], for example, they investigated that rapid urbanization would facilitate the increase in PM_2.5_ pollution from the global perspective. This reflects the fact that rapid urbanization and urban expansion may often be accompanied by increased population density, complex road traffic and high energy consumption, promoting an increase in PM_2.5_ emissions and thus aggravating PM_2.5_ pollution.

In Southern China, in particular, IO was also found to significantly impact the spatial heterogeneity of PM_2.5_ concentrations. Many studies have demonstrated that industrial activities were direct pollution source of PM_2.5_. For example, Wang et al. [26] and Yang et al. [27] demonstrated that IO and PM_2.5_ concentrations in China were associated with industrial emissions. Similarly, Zhang et al. [46] suggested that the proportion of the secondary industry, such as energy-intensive and high-polluting mining, construction, and manufacturing, is the most important factor driving PM_2.5_ pollution via direct and secondary reactions in China. However, Zhang et al. [35] showed that in the experiment of North China, no significant correlation was observed between IO and PM_2.5_. The difference may be due to that the difference in study area, as even the same factors can have different correlations with PM_2.5_ in different regions. Moreover, the greater impact of the interaction of UR and IO on PM_2.5_ revealed that the level of urbanization and industrial activities have significantly enhanced their effect on PM_2.5_ alone. Additionally, some regions of China lack appropriate control measures and have failed to implement effective energy-saving measures and environmental protection policies to tackle PM_2.5_ emissions. Due to rapid urban development, there has been a great increase in industrial activities, road density and energy consumption, which are, in turn, related to the direct source of PM_2.5_ pollution. Therefore, when formulating policies related to pollution prevention and control in the future, appropriate restrictions on highly polluting and emitting industrial activities should be considered in order to achieve sustainable development.

Most of the previous studies have also shown that temporal changes in PM_2.5_ pollution are related to meteorological factors, which have a certain impact on the accumulation and diffusion of pollutants [47,48]. Our results showed that AT showed the highest correlation with PM_2.5_ risk among meteorological factors in Northern and Southern China during the study period, which is similar to the results of other studies. Tai et al. [49] also found that temperature had the greatest and positive effect on PM_2.5_ concentration, validating our results. Chen et al. [50] showed that human activities and climate change can have some influence on the trend of submicron particle pollution. However, Chen et al. [23] found a negative relationship between PM_2.5_ and temperature in summer and autumn, which was different from the previous research. Notably, the interaction of AT and RH/VP had the largest effect on PM_2.5_ concentrations, both with *q* values of 0.76, indicating that the average temperature itself not only had a strong effect on PM_2.5_ concentrations, but also enhanced the effect of one factor on PM_2.5_ to a large extent when combined with other factors. The reason for this phenomenon could be that secondary aerosols are more likely to form if they are at higher temperatures, which would lead to an increase in PM_2.5_ [51]. In addition, if at lower temperatures, the use of heating and power generation systems can be greatly increased, leading to the emission of large amounts of pollutants, thus increasing PM_2.5_ pollution [52].

The highlight of this study suggested that the interactive effects between different socioeconomic and meteorological factors, relative to their individual effects, all enhance the impact on PM_2.5_ pollution. The interactive effects between socioeconomic and meteorological factors exacerbate the degree or extent of air pollution to some extent and play a mutually reinforcing role in terms of PM_2.5_ concentrations. Specifically, PM_2.5_ pollution is influenced by various factors, including meteorological, socioeconomic, anthropogenic emissions and so on. By analyzing the interaction effects of various factors, this study provided a new perspective for studying PM_2.5_ pollution, which can provide some scientific references for policy formulation. That is, when formulating policies, it is important to consider not only the influence of a single factor, but also the interaction of various factors on PM_2.5_ pollution to contribute to the improvement of environmental quality. In addition, China is a vast country with different levels of PM_2.5_ pollution in different regions, which can provide a reference for policy-making and improve public awareness and understanding of PM_2.5_ pollution. Since cities are common carriers of pollutants and their influencing factors, interactions can have a more considerable impact on the environment, weather, and human living conditions [53], because no factor exists alone. Therefore, in future policy formulation and pollution prevention and control, the interaction of factors should be considered comprehensively in order to achieve an integrated layout and targeted treatment for the region’s sustainable development.

The results of BSTHM presented high and low-risk regions of PM_2.5_ pollution. The high-risk regions are mainly located in North China and the YRD, indicating that these regions experience severe PM_2.5_ pollution. The low-risk regions are mainly located in the northeast and southwest regions, indicating that the PM_2.5_ level in these regions is relatively low. The results suggested that both meteorological and socioeconomic conditions had an intervening in PM_2.5_ pollution levels, which can be demonstrated from the previous studies. Zhang et al. [35] reported that in North China, the level of urbanization was positively correlated with PM_2.5_ pollution, i.e., the higher the level of urbanization, the more severe the PM_2.5_ pollution. The results of Yang et al. [27] showed that the industrial output presented as having a dominant effect on PM_2.5_ concentration. The underlying mechanism may be that with the rapid socioeconomic development, increased industrial activities, population gathering, and high road density, would promote the growth of PM_2.5_ concentrations.

There are some limitations that should be mentioned. First, PM_2.5_ pollution and impact factors (meteorological and socioeconomic factors) used in this study are spatially based on provinces and temporally based on years, which can bring some uncertainties to the results, and future studies can further improve on these aspects, more detailed spatial units will be used, such as cities. Second, although the accuracy of the raw PM_2.5_ data is quite high, there are also uncertainties in different regions. Therefore, using data with high spatial and temporal resolution in further studies makes the error in quantifying the effects of influencing factors on PM_2.5_ smaller.

## 5. Conclusions

We investigated the spatial–temporal heterogeneity of PM_2.5_ pollution across China between 2000 and 2018 using BSTHM and quantified the influence of meteorological and socioeconomic factors on PM_2.5_ in the northern and southern regions of China, respectively, using GeoDetector. Temporally, PM_2.5_ concentrations continued to increase during 2000–2008, followed by fluctuating trends during the early and late stages of the 2008 Beijing Olympics, and a decreasing trend in PM_2.5_ after the enactment of the APPCAP policy in 2013. The North China Plain and the Yangtze River Delta region are high-risk regions of PM_2.5_ pollution. Moreover, meteorological factors (especially temperature) and socioeconomic factors (especially population density) are closely related to the temporal and spatial heterogeneity of PM_2.5_ pollution, and there are regional differences. The above findings provided us with a clearer understanding of PM_2.5_ pollution patterns across Chinese provinces, which can be considered a scientific reference for implementing the air quality policies and providing guidance for remedial measures in some polluted provinces.

Notably, the contribution of this study is mainly to explain that there are different dominant factors affecting PM_2.5_ pollution within different regions and the interactions between each regional factor on PM_2.5_ pollution to provide scientific reference for policy formulation. The dominant socioeconomic factors are population density and industrial activities, indicating that with rapid urbanization, anthropogenic emissions and industrial activities would contribute to the rise of PM_2.5_ concentration and, therefore, industrial expansion, especially polluting industries, should be adequately limited during rapid urban development.

## Figures and Tables

**Table 1:** The *q* values (*q*_1_, *q*_2_) for the association between PM_2.5_ concentrations and meteorological factors in Northern and Southern China, respectively.

Meteorological Factors	*q*_1_	*q*_2_
Average temperature (°C)	0.64 **	0.51 **
Vapor pressure (hPa)	0.50 **	0.68 **
Precipitation (mm)	0.15 **	0.35 **
Relative humidity (%)	0.08 *	0.37 **
Wind speed (m/s)	0.08	0.13 **

Note: * statistical significance level = 0.05; ** statistical significance level = 0.01.

**Table 2:** The *q* values (*q*_1_, *q*_2_) for the association between PM_2.5_ concentrations and socioeconomic factors in Northern and Southern China, respectively.

Socioeconomic Factors	*q*_1_	*q*_2_
Population density (person/km^2^)	0.62 **	0.66 **
Non-agricultural proportion of the population (%)	0.37 **	0.27 **
Per capita GDP (10^4^ CNY)	0.13 **	0.12 **
Industrial output (10^4^ CNY)	0.10	0.36 **
Proportion of second industry (%)	0.07	0.33 **

Note: ** statistical significance level = 0.01.
